# DynamicRoots: A Software Platform for the Reconstruction and Analysis of Growing Plant Roots

**DOI:** 10.1371/journal.pone.0127657

**Published:** 2015-06-01

**Authors:** Olga Symonova, Christopher N. Topp, Herbert Edelsbrunner

**Affiliations:** 1 IST Austria, Klosterneuburg, Austria; 2 Donald Danforth Plant Science Center, St. Louis, Missouri, USA; University of Nottingham, UNITED KINGDOM

## Abstract

We present a software platform for reconstructing and analyzing the growth of a plant root system from a time-series of 3D voxelized shapes. It aligns the shapes with each other, constructs a geometric graph representation together with the function that records the time of growth, and organizes the branches into a hierarchy that reflects the order of creation. The software includes the automatic computation of structural and dynamic traits for each root in the system enabling the quantification of growth on fine-scale. These are important advances in plant phenotyping with applications to the study of genetic and environmental influences on growth.

## Introduction

Root system architecture plays a key role in plant fitness and crop productivity. Climate change, a growing global population, and unsustainable use of fertilizers are responsible for the pressing need to understand how root systems grow and interact with their environment, with the goal to develop robust and efficient crops [1–3]. Studying plant roots systems has been hindered by their complex three-dimensional branching topology, environmental growth plasticity, as well as the opacity of the soil. In recent years, however, various imaging technologies have been applied to monitor the formation and development of roots non-disruptively and throughout a time period: X-ray [4–8], MRI [9], PET-MRI [10], laser [11], and optical imaging [12, 13]. These technologies reconstruct the 3D shape of a root system and enable the assessment of traits that were impossible to quantify by hand measurements before [5, 12–15]. Most of the existing tools compute traits for static shapes, and if more than one shape representing the same root system is available, the global traits are computed without separating the contributions of individual branches [16]. Software that analyzes a time-series of 2D root images but requires significant user input to track growth has been introduced in [17, 18]. Several high-throughput platforms were developed for the analysis of video clips of growing dicot roots [19–21]. Advances in the fields of computer graphics and computer vision make it possible to quickly align large shapes that are at least partially similar [22, 23]. These methods can be used to improve the analysis of time-series but have not yet been exploited for plant phenotyping.

In this paper, we present the DynamicRoots software designed to automatically process a time-series of 3D reconstructions of a growing root system. It registers the 3D shapes in a common coordinate system and creates a data structure that records the growth. Using this data structure, we estimate dynamic traits of individual branches, which we aggregate to global traits. Our contributions to the state-of-the-art are:

- a geometric graph representation of a root system that reflects its hierarchical structure;
- an algorithm that guarantees temporal coherence of the hierarchical decomposition and computes the time function recording the growth process;
- a suite of structural and dynamic root traits including elongation rates and branching frequency.

The computed traits facilitate the analysis of root systems in unprecedented resolution of time and space. We illustrate the software by analyzing several time-series of reconstructed rice and maize roots.

**Outline**. The Methods section gives a detailed description of the algorithm that constructs the growth record for a time-series of 3D root shapes, and lists the structural and dynamic traits computed from this record. The Results section explains how we validate the software and presents experimental results for rice and maize root systems. We conclude the paper with a discussion of current limitations and future research directions.

## Methods

The goal of this work is to acquire the shape of a growing root system, to compactly represent it in a unified data structure, and to compute traits that reflect the structural and dynamic properties of the root system. We divide the process into five steps:

1. Take 2D images of root systems and construct a time-series of 3D shapes.
2. Align the shapes to define depth as the geodesic distance from consistently detected seed areas.
3. Use the depth function to decompose each shape into a hierarchy of branches.
4. Construct the time function and reorganize the hierarchy by repairing inconsistencies between depth and time of growth.
5. Use the time function on the branch hierarchy to compute structural and dynamic traits of the root system.

Correspondingly, we have five subsections explaining the steps in appropriate detail. Step 1 relies on prior work presented elsewhere, while the other four steps are novel contributions reported for the first time in this paper. Steps 2 to 4 construct the growth record, and Step 5 makes use of this unifying data structure.

### Background and Assumptions

We review the prior work to the extent necessary to provide the context for our work. To overcome the obstacle of opaque soil, we grow rice and maize plants in gel with nutrients inside transparent cylindrical containers [13], monitoring the development of the root system for several weeks. To construct the 3D shape of a root system, we place the container on a rotating table connected to a camera and a computer, and we take 40 images at 9° increments. Currently, 20 of these images are used to reconstruct the shape of the root system represented as a collection of voxels in 3D space [24]. Letting *t* be the time of the acquisition, we write 𝕍_*t*_ for the reconstructed shape. Depending on the purpose, we will think of 𝕍_*t*_ as a subset of ℝ^3^, a collection of voxels, or a graph whose nodes are the voxels and whose arcs connect neighboring voxels. Drawn as straight line segments ending at voxel centers, the arcs have length 1, $\sqrt{2}$, or $\sqrt{3}$ times the length of a voxel edge.

In order to analyze the growth of a root system, the same plant is reconstructed at different moments in time. We thus get a time-series of shapes 𝕍_*t*_, for *t* = 0,1, …, *T*, which provides a much richer source of information about the root system than a single such shape. However, harvesting this information is more difficult since we need to compare, quantify similarities and differences, and resolve occasional contradictions. To do this automatically within our software platform, we make use of the following three assumptions:

- A_1_: A root system grows in only two ways: by generating a new branch at a fork, and by elongating an existing branch at the tip.
- A_2_: A root system is connected and contractible. In other words, the branches do not form loops, and they do not surround voids in space.
- A_3_: The time-series is dense enough to observe the correct root hierarchy: when a side branch first appears at a fork, it is shorter than the parent branch.

While we have biological reasons to believe that A_1_ and A_2_ are true, both assumptions are sometimes violated by the reconstructed shapes. Our software can tolerate mild violations of all of the three assumptions but will fail when the reconstructed shapes contain gross contradictions to our expectations.

### Depth, Seed, and Alignment

In this and the next two subsections, we explain how to construct the *growth record* of a root system, which provides a decomposition into a hierarchy of branches together with the time of growth. As a first step, we equip each reconstructed shape with a function *φ*_*t*_;𝕍_*t*_ → ℝ that maps each voxel to its geodesic distance from a *seed area*, a concept we will explain shortly. We call this distance the *depth* of the voxel, and we call *φ*_*t*_ the *depth function* of 𝕍_*t*_. After fixing a set of voxels *S*_*t*_ ⊆ 𝕍_*t*_ as the seed area, we compute *φ*_*t*_ using Dijkstra’s shortest path algorithm [25]. Running this algorithm on the graph of voxels is straightforward, but the result crucially depends on the choice of *S*_*t*_, which is inspired by the biological idea that the root grows from a particular first location. It will be more important that the location of this area is consistent among all shapes in the time-series than to correctly identify the biological beginnings of the root system. We therefore compute *S*_*T*_, and propagate its location to the other shapes after computing an initial alignment of all shapes in the time-series. We will explain the alignment first and return to computing the seed area thereafter.

Note that Assumption A_1_ implies 𝕍_*s*_ ⊆ 𝕍_*t*_ for all 0 ≤ *s* ≤ *t* ≤ *T*. To align the two shapes, we can therefore use an algorithm that requires that the first shape be contained in the second. Our algorithm of choice is the *4-point congruent sets (4PCS) algorithm* described in [22]. To apply it, we think of 𝕍_*s*_ as a collection of points in ℝ^3^, namely the centers of its voxels. The algorithm selects four approximately coplanar points from 𝕍_*s*_ and searches for a congruent configuration of four points in 𝕍_*t*_. Let *M* be the matrix of the rigid motion that moves the 4-point configuration in 𝕍_*t*_ to that in 𝕍_*s*_. While the 4PCS algorithm is fast, the alignment it produces can often be improved, and we use the *iterative closest point (ICP) algorithm* originally described in [26] for this purpose. It computes a second matrix *M*′, and we set *M*_*st*_ = *M*′ ⋅ *M* so that *M*_*st*_(𝕍_*t*_) is aligned with 𝕍_*s*_. To align all *T* + 1 shapes in the time-series, it suffices to compute *T* rigid motions, and we choose to compute *M*_0*t*_ for *t* = 1,2, …, *T*. To align *V*_*t*_ with *V*_*s*_, we then use $M_{st}=M_{0s}^{-1}\cdot M_{0t}$. Suppose now that we have computed the seed area in the last shape, *S*_*T*_ ⊆ 𝕍_*T*_. Using the rigid motions, we get *S*_*t*_ ⊆ 𝕍_*t*_ as the set of voxels closest to *M*_*tT*_(*S*_*T*_), for 0 ≤ *t* ≤ *T*.

To detect the seed area in 𝕍_*T*_, we perform *principal component analysis* (PCA) on a small neighborhood of a radius *R* around every voxel. Covariance matrices with three similar eigenvalues indicate a similar spread of points along the principal axes, which in 3D space corresponds to a spherical neighborhood. We find the biggest connected component of voxels with roughly spherical neighborhoods and form the seed area by adding voxels with offset at most *R* from this component. For the results reported in this paper, we used *R* = 3 and we suggest that this parameter is set to a value from the interval [*r*,2*r*], in which *r* is the average radius of the branches. If the seed was not detected—for example when the set of voxels with roughly spherical neighborhoods is empty or larger than an allowed fraction of the total volume—we choose a voxel in the center of the topmost horizontal slice of the root system as the seed area.

### Branch Hierarchy

As a second step in the construction of the growth record, we decompose each shape in the time-series into branches, which we organize hierarchically. This decomposition is inspired by the path decompositions of trees used in the design of efficient data structures; see e.g. [27]. Each branch starts at its tip at the bottom and extends upward, ending right before its fork, which lies somewhere between the two ends of the parent branch; we therefore represent each branch by the triplet of these entities: ${𝔹}_{}^{i}$ = (Tip^*i*^, Fork^*i*^, Parent^*i*^). An exception is the topmost branch, which does not have a parent. Among all possible branch decompositions, we are interested in the one defined by the depth function.

To construct the decomposition, we traverse the voxels in the order of decreasing depth, adding an arc right after its second endpoint. With each visited voxel, we create a new component, and with each visited arc, we either form an additional connection within the component, or we merge two components into one. After visiting all voxels and arcs, they are part of a single component representing the entire root system, and we recover the branches and the relations between them by analyzing the history of events. To make this concrete, we equip each voxel, *v*, with a pointer to another voxel, *ρ*(*v*). In the beginning, each voxel points to itself. After completion of the algorithm, each voxel points to the tip of its branch, except for the tip itself, which points to the tip of the parent branch. A *tip* is therefore identified by having other voxels point to it, a *branch* is represented by its tip and consists in addition of all voxels pointing to this tip, and the *parent branch* is given by the pointer stored at the tip.

The only interesting event during the course of the algorithm is when it encounters an arc that connects two components. Let its endpoints be the voxels *u* and *v*, and write *p* = *ρ*(*u*) and *q* = *ρ*(*v*) for the corresponding tips, noting that *p* ≠ *q* by assumption. Assume without loss of generality that *q* is deeper than *p*: *φ*(*p*) < *φ*(*q*). If the difference in depth between *u* and *p* is smaller than some fixed threshold, then we declare the entire branch to be a part of the branch of *q* by updating the pointer: *ρ*(*p*) = *q*. Otherwise, we consolidate the branch of *p* by declaring *v* as its fork and the branch of *q* as its parent.

As mentioned earlier, each branch is represented by its tip, *p*, and consists of all voxels *u* with *ρ*(*u*) = *p*, together with *p*. To avoid possible confusions arising from the two different uses of the *ρ*-pointer, we save the pointer to the parent branch by setting Parent(*p*) = *ρ*(*p*), and thereafter set *ρ*(*p*) = *p* to indicate that it belongs to the branch it represents. In addition, we store a pointer Fork(*p*) to the fork in the parent branch. The thus linked branches form what we call a *branch hierarchy*. At this moment, the hierarchy is determined by depth: a branch has a tip that is necessarily less deep than the tip of the parent branch. Ultimately, we would like the hierarchy be determined by age. In the next section, we will explain how the hierarchy can be modified to achieve this goal.

### Time Function

We are now ready to compute the time function of the last shape in the time-series, *τ*;𝕍 → ℝ with 𝕍 = 𝕍_*T*_. Compressing the information of the time-series into a single shape, it maps every voxel of 𝕍 to the moment in time the voxel was created. The time function is piecewise constant, with possibly large steps in particular but not exclusively at forks. We note that the time function is the central piece of the growth record that enables the dynamic analysis of the root system.

To prepare the computation, we initialize the time function to *τ*(*v*) = *T* for every voxel *v* ∈ 𝕍. Traversing the other shapes in the time-series from back to front, we use the Euclidean distance between voxels to match the branches of 𝕍_*t*_ with those of 𝕍. It is therefore important that the shapes in the time-series are aligned and registered into a common coordinate system, which we henceforth assume. Specifically, for each tip in 𝕍_*t*_, we find the closest voxel *v* ∈ 𝕍, and we match the branch of the tip with the branch 𝕁 in 𝕍 that contains *v*. Finally, we set *τ*(*u*) = *t* for all voxels *u* of 𝕁 whose depth satisfies *φ*_*T*_(*u*) ≤ *φ*_*T*_(*v*).

After running the algorithm, the set of voxels *u* ∈ 𝕍 with *τ*(*u*) ≤ *t* would ideally be the set of voxels in 𝕍_*t*_, for every 0 ≤ *t* ≤ *T*. Of course, this will never be the case in practice, but there is a reason other than the inevitable noise in the system that may lead to discrepancies between the computed and the ideal time function, namely possible inconsistencies between the decompositions into branches between 𝕍_*t*_ and 𝕍. Instead of detecting inconsistencies between the branch decompositions, we repair the time function. Suppose *v* ∈ 𝕍 is a fork at which the classification into main branch and side branch is incorrect. By Assumption A_3_, we get a conflict between the depth and the time. Specifically, we observe neighbors *u* of *v* for which

$$\begin{array}{c} \varphi (u)<\varphi (v)\;\text{and}\;\tau (u)>\tau (v). \end{array}$$ (1)

**Fig 1:**
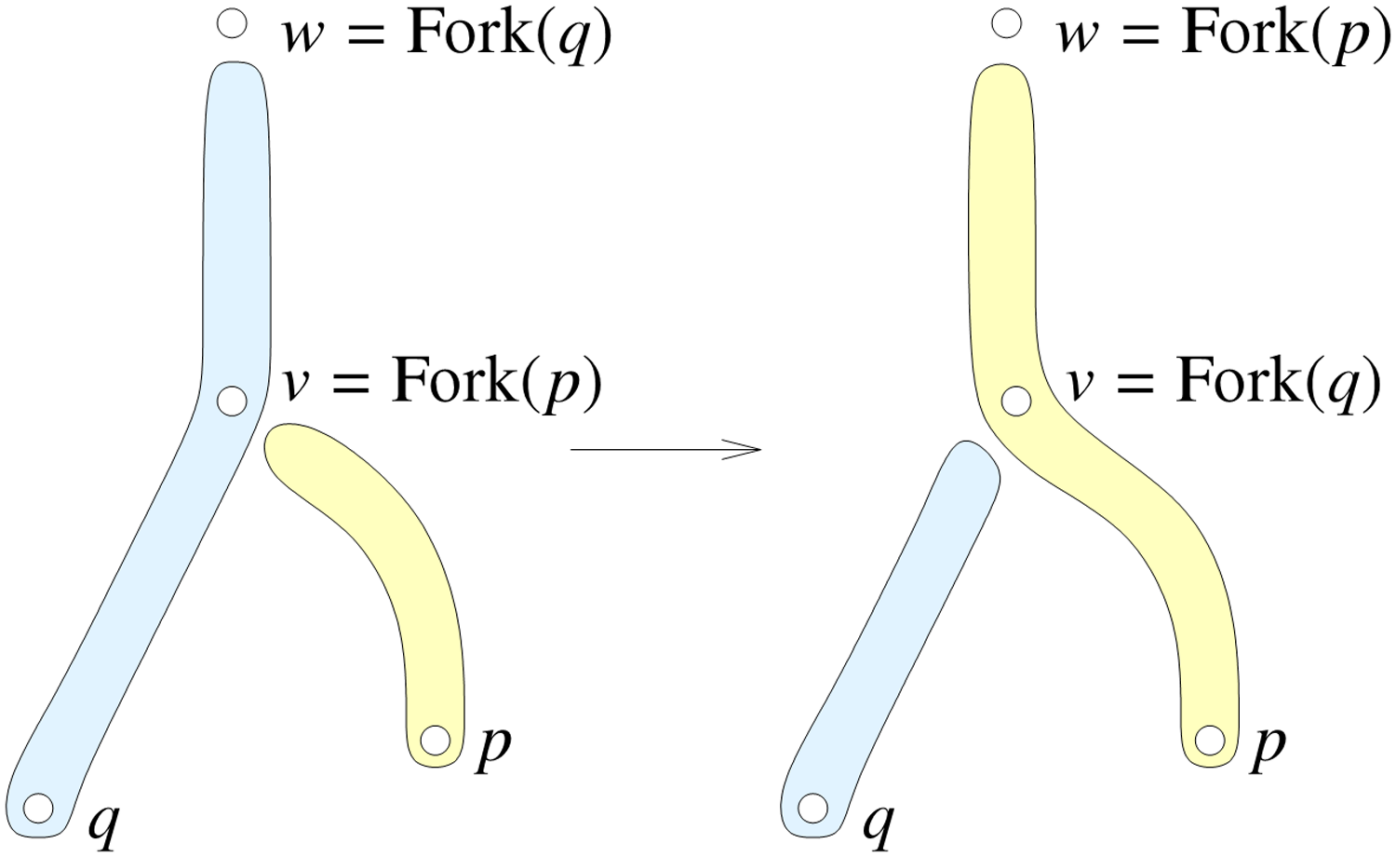
A switch at the fork *v*.

If we detect such a fork, we repair the inconsistency by an operation we call a *switch*. Letting *p* and *q* be the tips of the two branches at *v*, with *v* = Fork(*p*) but *v* ≠ *w* = Fork(*q*), we note that *q* = Parent(*p*). To perform the switch, we reassign the part of the branch of *q* between *v* and *w* to the branch of *p*; see Fig 1. In addition, the time at any voxel *u* within this part is set to the minimum of the current value and the value at *v*.

### Root Traits

Using the growth record explained above, we compute a suite of traits that describe a root system captured in a time-series. Denoting the growth record by 𝕍 = 𝕍_*T*_, we make essential use of the decomposition into branches, which we denote as ${𝔹}_{}^{i}$. Recall that ${𝔹}_{t}^{i}\subseteq {𝔹}^{i}$ is the sublevel set of the time function on the branch. We call the voxel $u\in {𝔹}_{t}^{i}$ with maximum depth the *tip* of the branch at time *t*. We compute the following traits for every branch ${𝔹}_{}^{i}$ and at every moment of time 0 ≤ *t* ≤ *T*:

- **volume**: the number of voxels in ${𝔹}_{t}^{i}$;
- **depth of tip**: the geodesic depth of the tip of ${𝔹}_{t}^{i}$;
- **location of tip**: the three Cartesian coordinates of the tip;
- **length**: the difference between the geodesic depth values of the tip and the fork;
- **switch event**: a logical variable that indicates whether or not ${𝔹}_{t}^{i}$ is longer than its parent branch;
- **tortuosity**: the ratio between the length of a branch and the Euclidean distance between tip and fork;
- **average radius**: the square root of the volume divided by *π* times the length;
- **angle to gravity**: the angle between the major PCA axis of ${𝔹}_{t}^{i}$ and the vertical direction;
- **angle to parent branch**: the angle between the major PCA axes of ${𝔹}_{t}^{i}$ and of its parent branch;
- **number of children**: the number of branches that have ${𝔹}_{t}^{i}$ as parent branch.

There is an ambiguity about which part of a branch should be taken into account when computing the root emergence angle or direction of growth. In order to avoid this uncertainty, we use the major PCA axis of the branch which shows the main direction of the spread of all the voxels forming this branch.

Only two of the measurements relate the root system to the surrounding, namely the location of the tip and the angle to gravity. Both are useful to study the reaction of the root system to nutrients and other local environmental conditions. The measurements we output for each branch can of course be accumulated to obtain overall traits of the root system, such as the **total volume**, the **total length**, the **total average radius**, and so on. Similarly, we can take differences to compute dynamic traits, as for example the **amount of growth** from time *t* to time *t* + 1. Beyond the traits listed above, we compute the following characteristics of the seed area:

- **seed volume**: the number of voxels in the seed area of the root system;
- **seed orientation**: the angle between the major PCA axis of the seed area and the vertical direction.

Comparing the seed volume with the total volume, we get the **volume fraction** of the seed area. The concrete representation of the seed area is useful in turning the geometric hierarchy of the branches into a biologically more meaningful one. The former is defined by assigning the index 0 to the topmost branch and increasing the index by 1 whenever we pass from a branch to a child branch. A biologically more meaningful classification is into

- *crown roots*, which emerge above the seed area;
- *seminal roots* with forks located within the seed area;
- the *primary root* corresponding to the topmost branch;
- *lateral roots*, which are the children of other branches.

**Fig 2:**
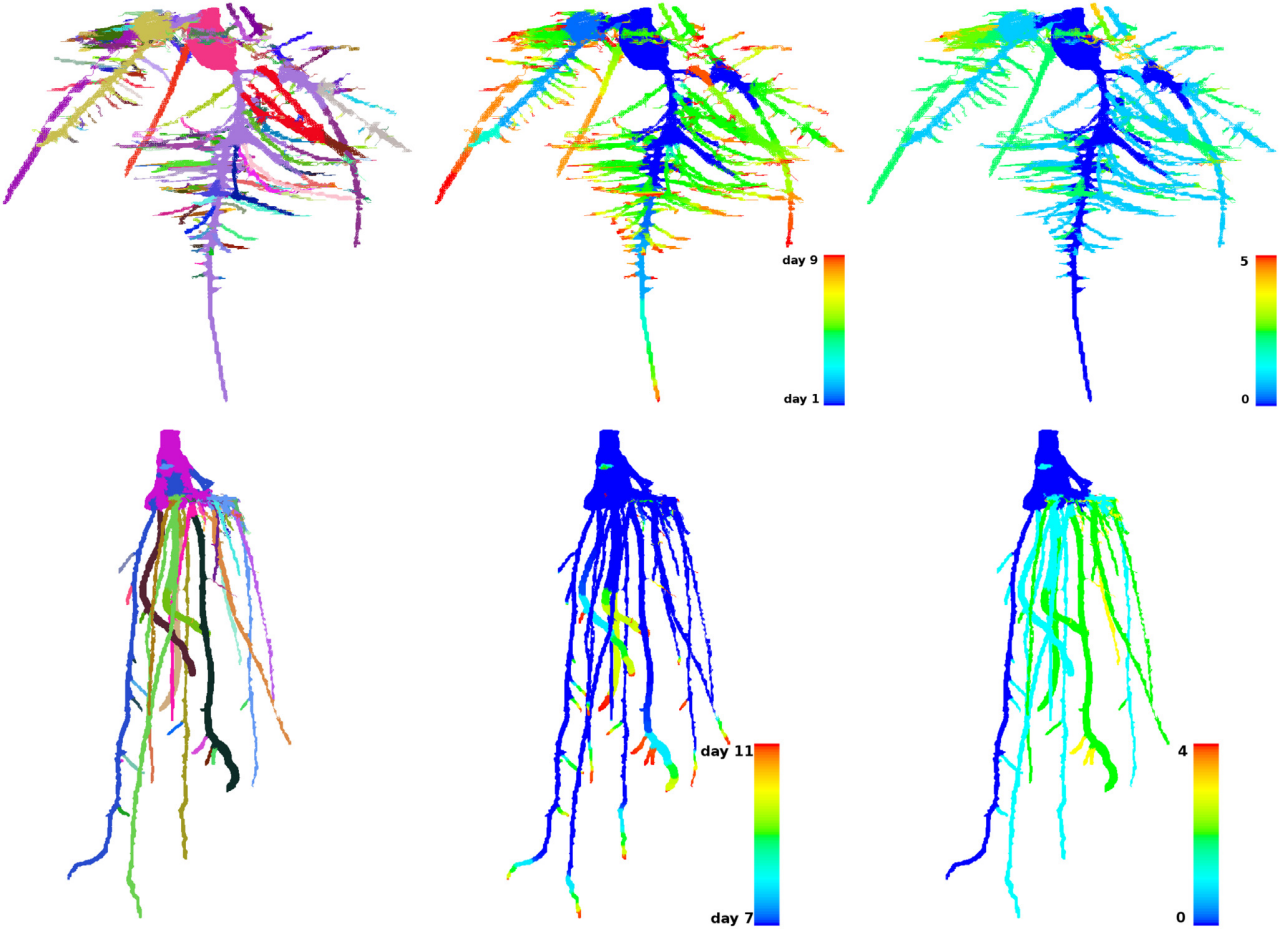
Structural and dynamical analysis of a root system. *Top row*: images of a maize plant imaged between the first and ninth days after planting. *Bottom row*: images of a rice plant imaged between the seventh and eleventh days after planting. *Left*: decomposition into a seed area and branches distinguished by color. *Center*: the color-coded time function. *Right*: the color-coded branch hierarchy.

Unfortunately, crown roots often emerge from the stem above the reconstructed space, which results in disconnected branches. The reconstruction software used in this study [24] enforces connectivity and tries to find the shortest path between the biggest component of the root system and a disconnected branch. This shortest path does not always join the branch in a biologically correct manner, making the classification into biological classes more challenging. At the moment, we explore other ways to differentiate between crown, seminal, and lateral roots. In particular, elongation rates might provide a good clue for detecting crown roots as their initiation is developmentally delayed but typically proceeds faster than of the other root types, see Fig 2.

## Results

Applying our methods, we obtain insights into the growth behavior of rice and maize plants. We note, however, that this is primarily a Methods paper, so we have limited the experiments to a few examples selected to illustrate useful features of the DynamicRoots software. The source code and the pre-compiled executable file for 64-bit Windows can be downloaded from http://dynamicroots.sourceforge.net/. The software has a command-line interface. In addition, we provide a python script, which organizes the input files and passes them as arguments to scripts that perform format conversion, data alignment, and time-series processing. The DynamicRoots software was developed in C++ using the Approximate Nearest Neighbor (http://www.cs.umd.edu/mount/ANN/) and ALGLIB (http://www.alglib.net/) libraries. DynamicRoots is an open source software distributed under the terms of the BSD license.

### Datasets and Validation

**Fig 3:**
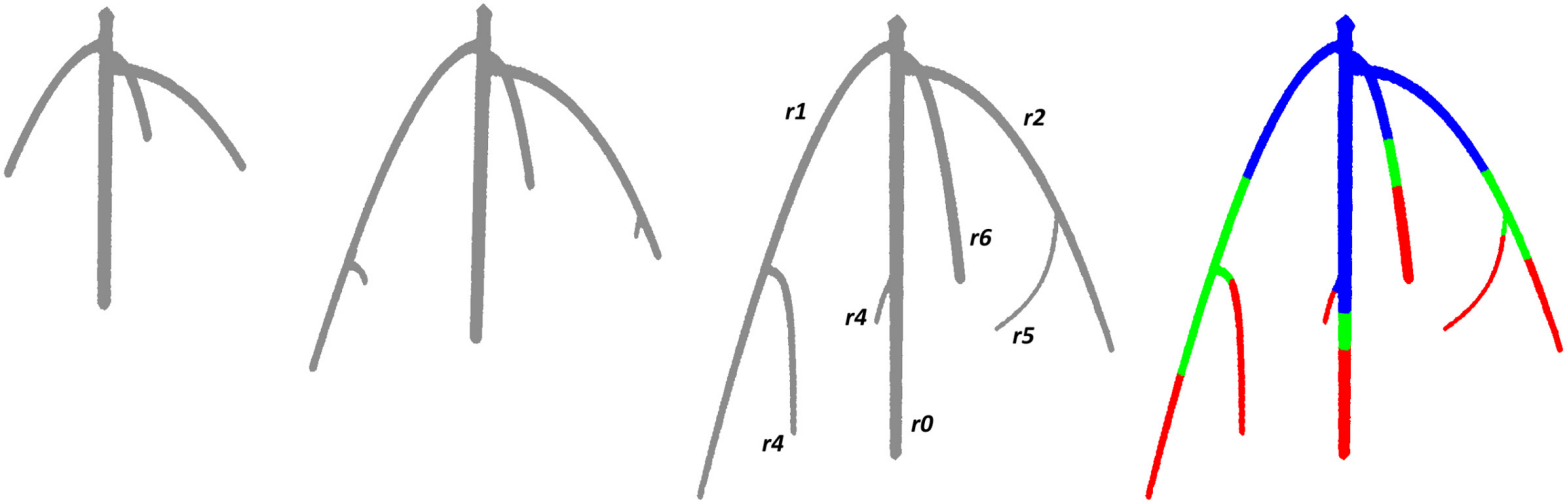
A series of three 3D models mimicking a growing root system designed to facilitate the validation of our software. The branches are labeled as in Table 1. The color-coded model at the far *right* illustrates the time function.

We use the software to analyze four different time-series of 3D shapes, three obtained by imaging growing rice and maize plants, and one artificially created to validate the software. The latter consists of three models forming a caricature of a growing root system; see Fig 3. Created from a collection of discretized algebraic curves, the models are obtained by sweeping balls of varying radii along the curves to get branches with prescribed thickness. Observe that the time-series satisfies Assumptions A_1_ to A_3_ stated in Section 1. After designing the models, we 3D-printed them from resin, imaged the three shapes, reconstructed the 3D shapes from the 2D images, and finally processed the result with our software. Table 1 compares the hand measurements of *branch length*, *depth of tip*, *volume*, *average radius*, and *tortuosity* with the values computed by our software. The agreement in branch length and depth of tip is remarkably high, with a somewhat larger relative error only for very short branches. The tortuosity of a branch depends on the estimation of its length and agrees fairly well with our hand measurements, with only 6% average relative error. The volume and therefore the average radius are slightly underestimated by the software because the reconstruction of the 3D shapes tends to lose some of the thickness due to small misalignments of the 2D images. Nonetheless, the total volume estimated by our software differs on average only by 5.2% from the volume computed as a product of the mass of the printed model and the resin density. In order to validate the values of the angle to parent branch, we represent the each branch as a line segment passing through the tip and the fork and compute the angle between them. We note that the software computes the angle between the branches using PCA and it is impossible to measure this value by hand. Nevertheless, we observe a similar behavior in the values measured by hand and computed with the software: the angles decrease as branches elongate. Unfortunately, there is no a common way to measure branch angles, and DynamicRoots offers a new approach to estimate this trait.

**Table 1:** Comparison of traits measured by hand and computed with DynamicRoots. Length, vertical depth, and radius are given in millimeters (mm), volume in cubic millimeters (mm^3^), angle in degrees, and tortuosity is dimensionless. The last column gives the average error of the DynamicRoots results compared to hand measurements.

Trait		Hand measurements			DynamicRoots results			Error
		*t* = 0	*t* = 1	*t* = 2	*t* = 0	*t* = 1	*t* = 2	
branch r0	Length	63.800	71.100	95.220	64.068	72.275	98.383	0.018
	Depth	63.800	71.100	95.220	62.083	69.683	95.348	0.016
	Volume	450.976	502.576	673.071	444.766	491.641	621.281	0.037
	Radius	1.500	1.500	1.500	1.488	1.473	1.418	0.027
	Tortuosity	1.000	1.000	1.000	1.030	1.040	1.030	0.033
	Angle to parent	0.000	0.000	0.000	0.000	0.000	0.000	0.000
branch r1	Length	35.770	76.110	102.680	36.198	84.020	112.870	0.072
	Depth	36.860	76.620	99.610	34.443	75.388	100.940	0.032
	Volume	112.375	239.107	322.579	96.219	203.297	248.516	0.174
	Radius	1.000	1.000	1.000	0.920	0.878	0.838	0.122
	Tortuosity	1.140	1.050	1.058	1.120	1.120	1.120	0.048
	Angle to parent	41.957	27.139	25.140	27.600	20.360	18.700	0.283
branch r2	Length	42.980	66.770	99.060	46.173	72.208	98.910	0.052
	Depth	36.530	55.890	78.230	32.820	53.085	74.885	0.065
	Volume	135.026	209.764	311.206	131.938	187.922	229.125	0.130
	Radius	1.000	1.000	1.000	0.953	0.910	0.858	0.093
	Tortuosity	1.075	1.136	1.237	1.120	1.160	1.180	0.036
	Angle to parent	57.645	49.580	40.981	55.200	43.570	36.840	0.088
branch r3	Length		7.000	37.550		4.335	39.833	0.221
	Depth		57.960	83.350		55.803	81.983	0.027
	Volume		17.224	92.395		8.516	54.703	0.457
	Radius		0.885	0.885		0.790	0.660	0.181
	Tortuosity		1.127	1.097		1.000	1.110	0.062
	Angle to parent		90.000	40.030		45.360	15.980	0.548
branch r4	Length			9.270			7.940	0.143
	Depth			66.480			65.203	0.019
	Volume			13.665			6.875	0.497
	Radius			0.685			0.525	0.234
	Tortuosity			1.150			1.070	0.070
	Angle to parent			38.432			15.49	0.597
branch r5	Length		4.300	27.830		3.538	32.415	0.171
	Depth		51.190	71.190		47.538	69.125	0.050
	Volume		6.339	41.025		2.484	19.609	0.565
	Radius		0.685	0.685		0.473	0.440	0.334
	Tortuosity		0.883	0.984		1.000	1.110	0.130
	Angle to parent		62.507	60.122		24.510	8.940	0.730
branch r6	Length	27.490	35.010	53.730	25.753	36.245	56.103	0.048
	Depth	30.260	36.970	54.370	24.633	33.623	50.628	0.115
	Volume	86.362	109.987	168.798	76.719	103.422	155.781	0.083
	Radius	1.000	1.000	1.000	0.975	0.953	0.940	0.044
	Tortuosity	1.180	1.113	1.130	1.070	1.100	1.110	0.041
	Angle to parent	49.957	46.420	35.918	43.190	36.200	27.890	0.193
total	Length	170.040	260.290	425.340	172.190	272.620	446.453	0.037
	Volume	784.738	1084.997	1622.737	749.641	997.281	1335.891	0.101
	Mass × Density	694.000	967.000	1397.00	749.641	997.281	1335.891	0.052

The first of the three natural datasets consists of 478 time-series of 3 shapes each, reconstructing root systems of growing rice plants on Days 12, 14, 16 after planting. Using static traits, this data has been used in [16] to study the genetic control of shape and development of the root architecture. We intend to extend this study with the dynamic traits and the information about the branch hierarchy available from our software. The second natural dataset consists of 4 time-series of 25 shapes each, reconstructing root systems of growing rice plants imaged every two hours from 8am to 4pm for five days starting from Day 7 after planting. The third natural dataset consists of 3 time-series of 23 shapes each, reconstructing root systems of growing maize plants images from Day 1 to Day 9 after planting.

The relatively high temporal resolution in the last two datasets leads to detailed characteristics of the root development not available in the first dataset.

### Traits

The traits described in Section 1 are output in a file for each time-series; see the supplementary material (S1 Table), which includes the file for the maize plant shown in the top row of Fig 2. For complete disclosure, our software also outputs files containing the color-coded geometry of the root system, representing the time function and the branch hierarchy, among other information; see Fig 2.

**Fig 4:**
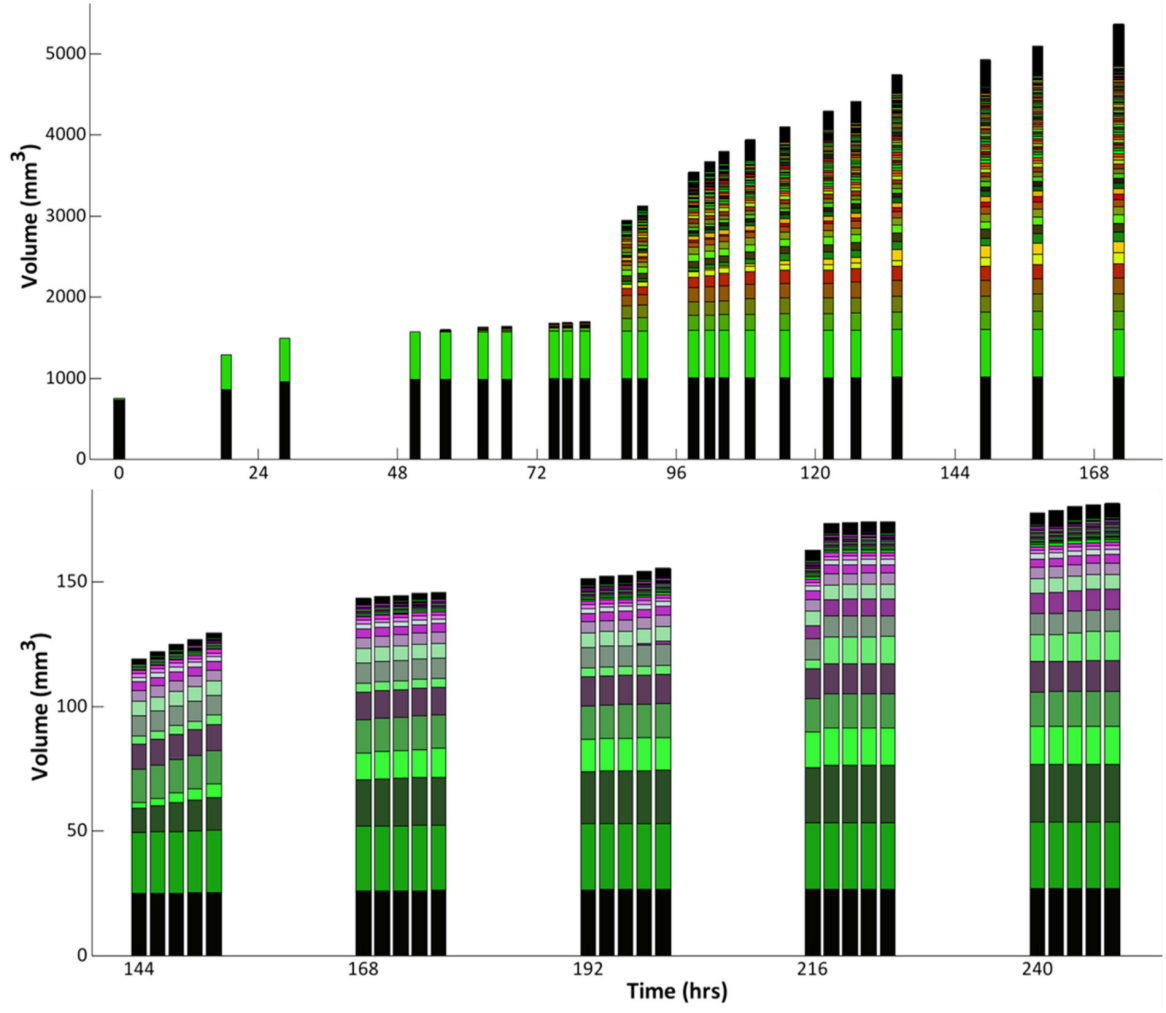
Volume dynamics of the root systems in Fig 2; maize at the *top* and rice at the *bottom*. The colored segments within each vertical bar show the volume contributed by individual branches.

We use the remainder of this subsection to demonstrate how DynamicRoots can be used to analyze growth dynamics. As a first example, we look at the dynamic addition of volume to the shapes for the two displayed time-series. Each vertical bar in Fig 4 represents the root system at a moment in time, with the colored segments representing branches in the decomposition. The displayed behavior reveals a remarkable dynamics in the growth process: the volume of the maize root almost doubles in the course of just a few hours, with the bulk of the additional volume contributed by newly formed branches (see Fig 4 top, between 72 and 96 hours).

**Fig 5:**
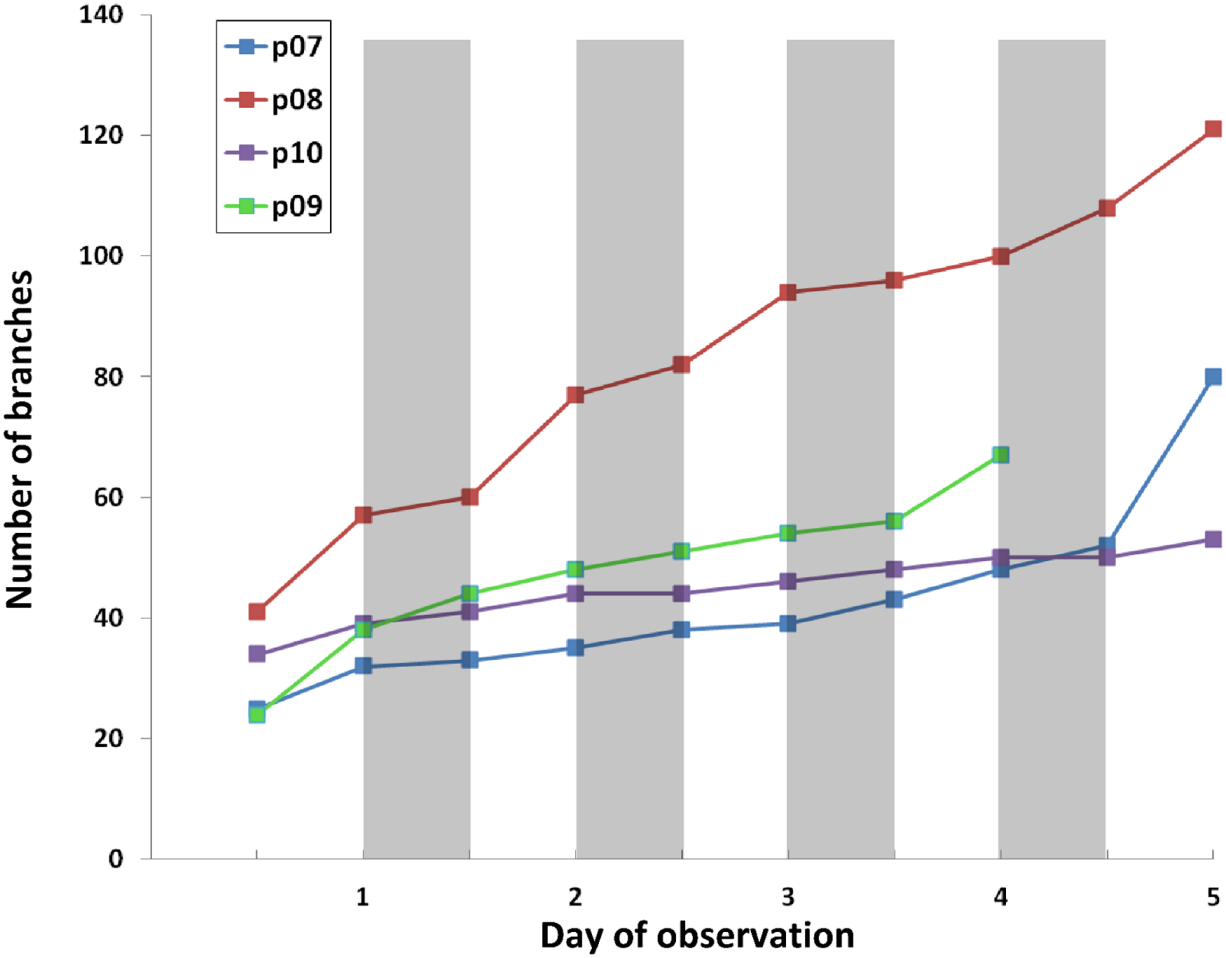
The branching dynamics of rice plants during five consecutive days. The shading distinguishes day- from night-time, highlighting that more branches are formed during day-time.

As a second example, we look at the branching dynamics, and in particular at the difference between the number of branches formed during day- and night-time. Fig 5 shows the number of branches as graphs over time for four rice plants. Observing the root systems during five consecutive days, we note that there are more branches formed during day-time than during night-time. The rice plants grew in the 12-hour cycle with the temperature of 28°C and 25°C at day and night. In [28] the authors studied diurnal elongation rates in *Arabidopsis thaliana* and observed higher elongation rates at night time than during the day. In [29] the authors demonstrated that growth conditions have a great impact on the diurnal growth patterns in sorghum and rice plants, they did not find differences in day-night elongation rates when the temperature was constant. In our data, we did not see differences in elongation rates at day and night time for plants in Fig 5. While more experiments are needed to understand the branching dynamics of root systems, it is clear that DynamicRoots can be of valuable assistance in collecting and analyzing the data.

### Performance

We distinguish between performance characteristics that pertain to the speed and the correctness of the software. In addition to the traits discussed above, the software outputs performance statistics, including the **running time**, the **average distance** between aligned 3D shapes, and the **number of switch events**. For example, the average running time for one time-series from our first dataset, including file format conversion, graphical file output, shape alignment and processing, is 51 seconds on an Intel Core 3.4GHz desktop computer.

We use the remainder of this subsection to illustrate how we make use of the performance measurements. A delicate step in our algorithm is the alignment of the 3D shapes in a time-series. We underline the fact that the alignment performs quite accurately due to the property of the 4PCS algorithm which can robustly align shapes even with small overlap, noise, and outliers [22]. To shed light on the quality of the alignment, we compute the average distance between the aligned shapes and provide the result as an indicator of the quality of the achieved alignment. More precisely, we sample 10% of the voxels in the first shape, 𝕍_0_, and we compute the Euclidean distance to the closest voxels in 𝕍_1_ to 𝕍_*T*_. For a high quality alignment, the average distance will be small, namely less than the edge length of a voxel. In the first dataset, there are several time-series for which the accuracy is compromised by technical difficulties with the imaging platform. For these series, the average distance we observe is significantly larger than for series that do not suffer from these imaging artefacts: e.g. 5.03 versus 0.84 times the length of an edge. In our analysis of the first dataset, we have used a threshold of 10.00 edge lengths for the average distance of the alignment beyond which a shape was excluded from the computations.

Another measurement that varies significantly with the quality of the alignment is the number of switch events. On average, we observe 9.04 switches between shapes in the first dataset. However, in the time-series affected by the mentioned image artefacts, this number increases to 58.96. The number of switches by itself can be an interesting biological trait as it measures how often a branch out-grows its parent branch. However, if the number is significantly higher than the average, then this may be reason for concern since it might be caused by technical difficulties in the acquisition pipeline rather than biological reality.

## Discussion

The DynamicRoots software provides novel capabilities for the analysis of growing root systems of plants. The analyzed shapes come from plants grown in a gel substrate, but can be derived from any growth and imaging system. A logical next step is the genome-wide analysis of dynamic traits for root systems of agricultural plants. Given the availability of the decomposition into branches and the detailed per-branch information, it is time to study the local and global response of plants to localized nutrients in the environment.

While we hope that our software finds many users and contributes to our understanding of root systems, it is important to point out that there are pitfalls in using it. Most root systems we considered have 3D reconstructions with touching branches that lead to violations of our Assumption A_2_. As a consequence, there are loops in the reconstructed shape which leads to faulty decompositions into branches. Fortunately, such mistakes are local and do not affect branches away from the loops. Ideally, the software would have the built-in capability to remove loops by separating touching branches, but this is a challenging problem that awaits a satisfactory solution.

Nonetheless, the framework described here represents a major advance in root phenotyping methodology. For the first time, the growth dynamics of each branch in a complex three-dimensional root system can be automatically tracked and quantified over time. Thus, DynamicRoots will enable high-throughput analysis of local growth response to a wide-range of biotic and abiotic plant-environment interactions, as well as provide a powerful tool to answer fundamental questions such as: “How is root growth regulated over the circadian clock?”, and “How do local growth decisions of each root tip contribute to global root architecture?”.

## Supporting Information

S1 Table Root traits computed by DynamicRoots for the maize root shown in Fig 2. The names and values of the traits are listed in columns, rows correspond to individual branches.
(TXT)
